# Efficacy of Contact Lens-Based Interventions in Slowing Myopia Progression in Children: A Systematic Review

**DOI:** 10.7759/cureus.94217

**Published:** 2025-10-09

**Authors:** Abdulaziz A Alagsam, Hani A Al-Ghamdi, Rola M Alradaddi, Linan M Khormi, Farah T Alfuhigi, Majd A Alhazmi, Haya D Alshutayli, Yara M Alqahtani, Ghadi A Althobaiti, Muath I Masmali, Majed M Rufidi

**Affiliations:** 1 Ophthalmology, Prince Mohammed bin Nasser Hospital, Jazan, SAU; 2 College of Medicine, Al-Baha University, Al-Baha, SAU; 3 College of Medicine, Qassim University, Buraydah, SAU; 4 College of Medicine, Jazan University, Jazan, SAU; 5 College of Medicine, Jouf University, Sakaka, SAU; 6 College of Medicine, King Saud bin Abdulaziz University for Health Sciences, Riyadh, SAU; 7 College of Medicine, Majmaah University, Majmaah, SAU; 8 College of Medicine, Najran University, Najran, SAU; 9 College of Medicine, Taif University, Taif, SAU; 10 College of Medicine, King Khalid University, Abha, SAU

**Keywords:** axial length, childhood myopia, contact lenses, dual-focus lenses, extended depth-of-focus, multifocal lenses, myopia, myopia control, orthokeratology, spherical equivalent refraction

## Abstract

Myopia prevalence is increasing globally, with early-onset childhood myopia carrying a higher risk of progression to high myopia and associated sight-threatening complications. Contact lens-based interventions, including dual-focus, multifocal, extended depth-of-focus (EDOF), and orthokeratology designs, have been developed to slow progression, but their efficacy and safety remain variably reported. This systematic review aimed to evaluate the efficacy and safety of contact lens interventions in children, focusing on changes in spherical equivalent refraction (SER), axial length (AL), visual performance, compliance, and adverse events. A comprehensive search of PubMed, Web of Science, Scopus, and Cochrane Central Register of Controlled Trials (CENTRAL) was conducted from inception to September 10, 2025, following Preferred Reporting Items for Systematic Reviews and Meta-Analyses (PRISMA) guidelines, with eligible studies including randomized controlled trials and prospective comparative studies in children (<18 years) with myopia, compared with single-vision lenses or spectacles. Data extraction and quality appraisal were performed independently by two reviewers using the modified Downs and Black checklist. Of 8,036 records identified, 14 studies met the inclusion criteria. Dual-focus and high-add center-distance multifocal soft lenses (e.g., MiSight (CooperVision, Inc., San Ramon, California, United States), Biofinity+2.50 D (CooperVision, Inc., San Ramon, California, United States)) consistently reduced SER progression by ~0.40-0.73 D and AL elongation by ~0.23-0.32 mm over two to three years, corresponding to 40-60% relative slowing. EDOF and alternative designs (e.g., Esencia (Mark’ennovy Personalized Care, S.L., Madrid, Spain), MYLO (Mark’ennovy Personalized Care, S.L., Madrid, Spain)) showed variable but generally favorable effects, with short-term contralateral studies indicating efficacy comparable to MiSight. Orthokeratology reduced AL elongation by ~40% in early years, with cumulative long-term benefit (~0.69 mm less over 11 years), though evidence was limited by nonrandomization and attrition. Visual acuity was largely preserved, with small reductions in low-contrast performance (<1 line) that were not clinically significant. Efficacy was strongly adherence-dependent, particularly for high-defocus designs. No vision-threatening adverse events were reported; safety profiles were dominated by mild, self-limited ocular events similar to single-vision comparators. Overall, contact lens interventions, particularly dual-focus and high-add multifocal soft lenses, are effective and safe for slowing childhood myopia progression, with EDOF and orthokeratology also providing meaningful benefits. However, evidence quality and long-term durability vary. Consistent daily wear and early initiation appear critical for maximizing outcomes, supporting the clinical integration of contact lens-based myopia control strategies in pediatric practice while highlighting the need for further head-to-head and long-term comparative studies.

## Introduction and background

Myopia has emerged as a major public health challenge of the 21st century, with prevalence rising rapidly worldwide. Epidemiological data suggest that nearly half of the global population may be myopic by 2050, with particularly high rates in East Asia [1]. Early-onset childhood myopia is of particular concern, as younger children progress more rapidly and have a higher lifetime risk of developing high myopia [2,3]. High myopia is associated with sight-threatening complications, including myopic maculopathy, retinal detachment, glaucoma, and cataracts, contributing substantially to visual impairment and blindness [4].

Given the progressive nature of myopia, strategies to slow its progression during childhood are a major focus of vision science and clinical practice. Interventions in this critical period can reduce the final degree of myopia in adulthood, mitigating long-term risks [5,6]. Optical and pharmacological approaches have shown varying efficacy. While low-dose atropine is effective, side effects and limited acceptance in some populations highlight the need for alternative non-pharmacological strategies. Among these, contact lens-based interventions have gained increasing attention as a viable option [7-9].

Specialized contact lenses aim to alter peripheral defocus and slow myopia progression. Designs include dual-focus and multifocal soft lenses, extended depth-of-focus (EDOF) lenses, and orthokeratology. Daily disposable dual-focus lenses, such as MiSight (CooperVision, Inc., San Ramon, California, United States), reduce myopia progression by ~50-60% compared to single-vision controls [4,5,8]. Orthokeratology, which reshapes the cornea overnight, also significantly slows axial elongation over short- and long-term follow-up. Newer designs, such as EDOF and highly defocused concentric ring lenses, are under investigation, offering potential alternatives with distinct optical mechanisms [10].

Despite promising results, the literature on contact lens interventions is heterogeneous in study design, sample size, lens type, outcome measures, and follow-up duration [1,2,5,10]. Some trials show substantial efficacy, while others report modest or nonsignificant effects. Variations in compliance, baseline refractive error, ethnicity, and study protocols further complicate interpretation. Additionally, the long-term safety of these interventions in children requires careful evaluation. A systematic appraisal is therefore needed to clarify efficacy and safety, guide clinical practice, and identify research gaps.

This systematic review aims to synthesize evidence on the efficacy of contact lens interventions in slowing childhood myopia progression, focusing on dual-focus, multifocal, EDOF, and orthokeratology lenses. Key outcomes include spherical equivalent refraction (SER) and axial length (AL) changes, with secondary outcomes of visual acuity, quality of life, compliance, and safety. By consolidating findings from high-quality trials and prospective studies, this review provides an evidence-based understanding of contact lenses in pediatric myopia management.

## Review

Methodology

Literature Search Strategy

This review followed the Preferred Reporting Items for Systematic Reviews and Meta-Analyses (PRISMA) guidelines [11]. A comprehensive search was conducted in PubMed, Web of Science, Scopus, and the Cochrane Central Register of Controlled Trials (CENTRAL) from inception to September 10, 2025. The search combined controlled vocabulary (MeSH/Emtree) and free-text keywords for population (child, adolescent, pediatric, school-aged, juvenile), condition (myopia, near-sightedness, short-sightedness), interventions (contact lens, soft lens, multifocal, bifocal, dual-focus, concentric ring, extended depth-of-focus, orthokeratology, ortho-K), and outcomes (disease progression, axial length, spherical equivalent refraction, refractive error). Boolean operators (AND/OR) were used, and search strategies were tailored for each database. Filters limited results to human studies in English. Reference lists of included studies were manually screened to identify additional relevant articles.

Eligibility Criteria

Eligibility was defined using the Population, Intervention, Comparator, Outcomes, Study design (PICOS) framework [12]. Included studies involved children and adolescents (<18 years) with myopia, evaluated contact lens interventions (dual-focus, multifocal, EDOF lenses, or orthokeratology) aimed at slowing myopia progression, compared the intervention with single-vision lenses, spectacles, or no intervention, and reported at least one relevant outcome such as SER progression or AL elongation. Secondary outcomes included visual acuity, quality of life, compliance, and adverse events. Only randomized controlled trials, prospective cohort studies, or controlled clinical trials published in peer-reviewed journals were included. Exclusion criteria were studies on adults or mixed-age populations without pediatric data, non-contact lens interventions, non-comparative designs, abstracts without full text, reviews, case reports, or non-English publications.

Study Selection

Two reviewers independently screened titles and abstracts using the predetermined eligibility criteria. Full texts of potentially relevant studies were assessed for inclusion. Discrepancies at any stage were resolved through discussion with a third reviewer until consensus was reached.

Data Extraction

Data extracted from each study included author, year, country, study design, participant characteristics (sample size, age, sex, baseline refraction, AL), type of contact lens intervention, comparator details, wear schedule, follow-up duration, primary outcomes (SER and AL changes), secondary outcomes (visual acuity, quality of life, compliance, adverse events), and measurement tools. Extraction was performed independently by two reviewers, with conflicts resolved by a third reviewer.

Quality Appraisal

Methodological quality was assessed independently by two reviewers using the Downs and Black checklist [13], covering reporting, external validity, internal validity (bias and confounding), and power. Studies were classified as excellent (26-28), good (20-25), fair (15-19), or poor (≤14). Disagreements were resolved through discussion until a consensus was reached.

Results

Study Selection

**Figure 1:**
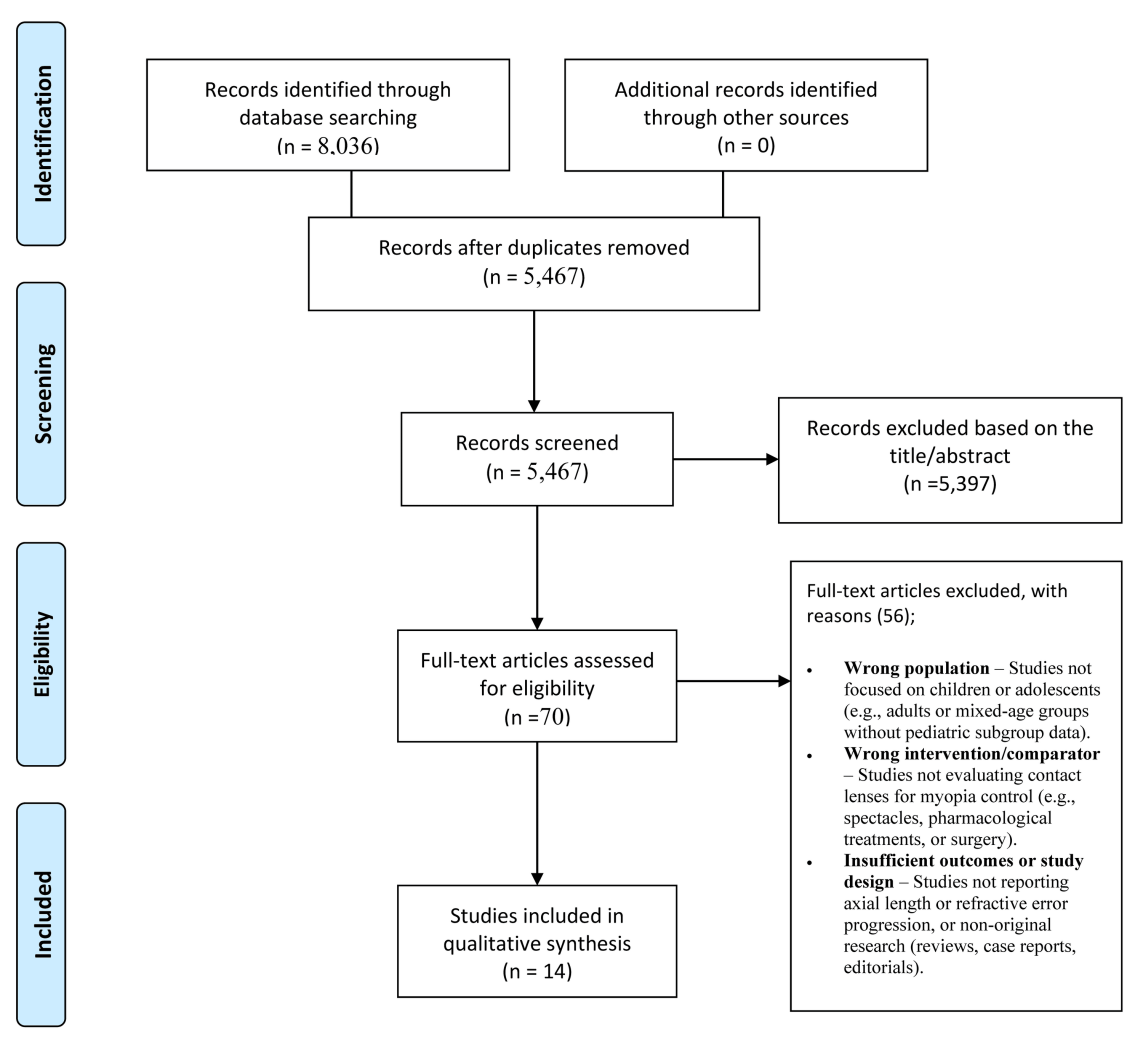
PRISMA flow diagram of study selection process PRISMA: Preferred Reporting Items for Systematic Reviews and Meta-Analyses

A total of 8,036 records were identified through PubMed (n = 1,378), Cochrane Library (n = 391), Scopus (n = 3,478), and Web of Science (n = 2,789). After removing duplicates, 5,467 records were screened by title and abstract, excluding 5,397 that did not meet the inclusion criteria (Figure 1). Seventy full-text articles were assessed, with 56 excluded for reasons including adult populations, non-contact lens interventions, irrelevant outcomes, or unsuitable study designs. Ultimately, 14 studies [1-10,14-17] met the inclusion criteria and were included in the qualitative synthesis.

Study Characteristics

Included studies enrolled school-aged children (six and 15 years) with baseline myopia of −1.5 to −3.5 D and ALs of ~24-25.5 mm. Interventions included daily disposable dual-focus or multifocal soft lenses (MiSight [14], Esencia (Mark’ennovy Personalized Care, S.L., Madrid, Spain) [5], Pegavision multifocal (Pegavision Corporation, Taoyuan City, Taiwan) [4]), monthly center-distance multifocals (Bifocal Lenses In Nearsighted Kids (BLINK) trials [7,10,15]), EDOF lenses (MYLO (Mark’ennovy Personalized Care, S.L., Madrid, Spain) [6], experimental EDOF [16]), high-defocus concentric lenses (DISC3.5plus [9]), and overnight orthokeratology [2,3]. Comparators were typically single-vision contact lenses or spectacles, with follow-up ranging from 12 months [8] to 36 months [7,14] and up to 11 years in orthokeratology extensions [2,3]. Outcomes included cycloplegic autorefraction, optical biometry, visual acuity, comfort, compliance, and safety (Table 1).

**Table 1:** Summary of clinical trials and prospective studies on myopia control interventions in children This table summarizes clinical trials and prospective studies evaluating contact lens–based or spectacle-based interventions for myopia control in children. AL: axial length; SER: spherical equivalent refraction; VA: visual acuity; QoL: quality of life; AEs: adverse events; SCL: soft contact lens; DFSCL: dual-focus soft contact lens; EDOF: extended depth of focus lens; SV: single-vision lens; PAL: progressive addition lens; OK: orthokeratology lens; BL: baseline; h: hours; mo: months; BC: base curve; D: diopter; SiHy: silicone hydrogel; DK: oxygen permeability; PCI: partial coherence interferometry; SS-OCT: swept-source optical coherence tomography; IOLMaster: optical biometer; ETDRS: Early Treatment Diabetic Retinopathy Study charts; CS: contrast sensitivity; fovea: central retina; VCD: vitreous chamber depth; CI: confidence interval; ITT: intention-to-treat; Ctrl: control group; Exp: experimental group. All values are reported as mean ± standard deviation unless otherwise specified. Menicon Z Night: Menicon Co., Ltd., Aichi Prefecture, Japan; MiSight: CooperVision, Inc., San Ramon, California, United States; Pegavision: Pegavision Corporation, Taoyuan City, Taiwan; IOLMaster 700: Carl Zeiss Meditec AG, Jena, Germany; Nidek ARK-1: NIDEK Co., Ltd., Gamagori, Aichi, Japan; Oculus Pentacam: Oculus Optikgeräte, GmbH, Wetzlar, Germany; MYLO: Mark’ennovy Personalized Care, S.L., Madrid, Spain; Topcon TRK-2P: Topcon Corporation, Tokyo, Japan; Biofinity: CooperVision, Inc., San Ramon, California, United States; Lenstar LS 900: Haag-Streit Diagnostics, Köniz, Switzerland; Kodak lenses: Eastman Kodak Company, Rochester, New York, USA; Grand Seiko WAM-5500: Grand Seiko Co., Ltd., Higashiosaka, Osaka, Japan; Aquamax: Mark’ennovy Personalized Care, S.L., Madrid, Spain

Study	Country	Study Design	Population Characteristics (Sample size, Age, Sex)	Baseline SER (D)	Baseline AL (mm)	Intervention (lens type, brand, parameters)	Comparator	Wear Schedule / Duration	Follow-up (months)	Outcomes & Measurement Tools (Primary: AL & SER change; Secondary: VA, QoL, compliance, AEs; Tools for AL/refraction)	Authors’ Conclusions
Anstice & Phillips [1]	New Zealand	Prospective, randomized, paired-eye control, investigator-masked, cross-over trial	N=40 children, 11–14 yrs, mean SER −2.71 ± 1.10; healthy, progressing myopes; ≤1.25 D astigmatism; balanced by sex/ethnicity	−2.71 ± 1.10	Not reported; progression measured	Dual-focus (DF) SCLs (Hioxifilcon A, daily wear, lathe-cut, 8.5 mm BC, 14.2 mm DIA; central correction + concentric +2.00 D defocus zones)	Single-vision distance SCLs (identical material/design without treatment zones)	Daily wear, ~12–13 h/day; DF lens in 1 eye, SVD lens fellow eye; swapped after 10 mo	20	Primary: SER (cycloplegic autorefraction Humphrey HARK-599). Secondary: AL (IOLMaster PCI), VA, contrast sensitivity, accommodation, pupil size, corneal power, compliance diaries	Period 1: DF eyes progressed −0.44 ± 0.33 D vs SVD −0.69 ± 0.38 D (37% reduction, p<0.001); AL growth 0.11 ± 0.09 mm vs 0.22 ± 0.10 mm (49%). Period 2: similar reductions. 70% children ≥30% slower progression, 50% ≥50% reduction. VA & CS unchanged. Mild handling issues; no serious AEs. DF lenses are effective.
Santodomingo-Rubido et al. [3]	Spain	Prospective, non-randomized, longitudinal comparative study (MCOS extension)	Initially N=61 (OK=31, SV=30); at 7 yrs: OK=14, CT=16; Age 6–12 yrs; healthy White children	OK: −2.27 ± 0.31; CT: −2.16 ± 0.26	OK: 24.39 ± 0.23; CT: 24.08 ± 0.27	Orthokeratology (Menicon Z Night, overnight wear)	Single-vision spectacles (initial 2 yrs), then SV or SCL (CT group)	Overnight lens wear; SV/SCL full-time	84	Primary: AL (IOLMaster), SER (cycloplegic autorefraction). Secondary: Corneal topography, corneal power/shape, ocular health, AEs	OK slowed AL elongation ~33% vs CT at 7 yrs (25.30 vs 25.43 mm). Stronger effect at 12–24 mo (40–42%, p<0.05), diminished by year 7 (p=0.062). Refractive progression is lower in OK (−0.29 D) vs CT (−5.00 D). No significant AEs after initial 2 yrs. Long-term efficacy confirmed.
Lee et al. [4]	Taiwan	Prospective, randomized, double-masked, parallel-group RCT	N=115 enrolled, 100 completed (55 boys, 60 girls), Age 8–15 yrs	Exp: −2.55 ± 1.35; Ctrl: −2.12 ± 1.26	Exp: 24.75 ± 0.94; Ctrl: 24.38 ± 0.89	Pegavision daily disposable multifocal SCL (design details not fully disclosed)	MiSight dual-focus SCL (CooperVision)	Daily disposable, ≥8 h/day, 7 days/week; monitored via diary/app	12	Primary: SER (cycloplegic autorefractor KR-8800; handheld retinoscopy), AL (IOLMaster). Secondary: High/low contrast VA, near VA, corneal curvature, ocular health, tear quality, AE monitoring	After 1 year, Pegavision (−0.50 D, +0.24 mm) and MiSight (−0.48 D, +0.22 mm) groups showed no significant differences. Both safe; mild corneal staining (4.1%). Similar efficacy and safety.
García-del Valle et al. [5]	Spain (multicentre: Madrid, Andalucía, Murcia)	Randomised, parallel, double-masked clinical trial	N=70 randomized (Study=36, Control=34); analyzed: Study=32, Control=26; Age 7–15 yrs (mean 12.2 ± 2.2); Sex: Study 19F/13M, Control 18F/8M	Study: −2.80 ± 1.79; Control: −3.31 ± 1.76	Study: 24.54 ± 0.89; Control: 24.48 ± 0.78	Esencia lens (Eurolent, custom-made SCL, hioxifilcon B, 50% water, progressive multifocal +2.00 D peripheral add, reverse geometry)	Conventional lathe-cut SCL (same material, aspheric single vision)	Daily disposable, quarterly replacement; monitored at baseline, 6, 12 months	12	Primary: Change in SER (cycloplegic autorefraction), AL (IOLMaster 700). Secondary: Corneal power, comfort, quality of vision, lens fitting, AEs (slitlamp). Tools: Nidek ARK-1, IOLMaster 700, Oculus Pentacam, slitlamp, questionnaires	Esencia lens slowed myopia progression significantly vs control: 51% less SER progression (−0.28 vs −0.57 D, p=0.02) and 41% less AL growth (0.13 vs 0.22 mm, p=0.03). Comfort, quality of vision, and fitting are similar. No serious AEs. Concluded effective and safe short-term.
Díaz-Gómez et al. [6]	Spain	Prospective, non-randomized comparative clinical trial	N=90 (CL=45, SV=45); Age 6–13 yrs; CL mean 10.9 ± 1.6, SV 11.2 ± 1.1; Sex ~20M/25F per group; parental myopia varied	CL: −2.80 ± 1.80; SV: −2.69 ± 0.99	CL: 24.62 ± 0.99; SV: 25.62 ± 0.87	MYLO EDOF CLs (Mark’ennovy, SiHy Filcon 5B, monthly, EDOF +1.50 D defocus; DK 60; UV filter)	Single-vision distance spectacles	Daily wear, ≥10 h/day, 6–7 days/week; monthly replacement; SV spectacles full-time for controls	24	Primary: AL (IOLMaster 700), SER (cycloplegic autorefraction Topcon TRK-2P). Secondary: High-contrast VA (logMAR), contrast sensitivity (Pelli–Robson), comfort & vision (questionnaire 1–10), slitlamp ocular health	After 24 mo: CL group −0.62 D SER & +0.37 mm AL vs SV −1.13 D & +0.66 mm (p<0.001). CARE 0.29 ± 0.06 mm; 53% CL ≤0.50 D progression vs 1% SV; 100% CL ≤0.50 mm AL vs all >0.50 mm SV. Slight VA reduction (<1 line), CS unaffected. High comfort, mild staining, no serious AEs. MYLO EDOF CLs are effective and safe.
Walline et al. [7]	USA (Ohio State & Univ. of Houston)	Multicenter, double-masked, randomized clinical trial	N=294 (7–11 yrs; mean 10.3 ± 1.2; 60% female; −0.75 to −5.00 D, <1.00 D astigmatism; VA ≥20/25). Randomized: High add=98, Medium add=98, SV=98	−2.39 ± 1.00	~24.5	Biofinity center-distance multifocal SCLs (Comfilcon A; High +2.50 D, Medium +1.50 D)	Biofinity single-vision SCLs	Daily wear, monthly replacement; encouraged ≥10 h/day	36	Primary: SER (cycloplegic autorefraction). Secondary: AL (Lenstar LS900), VA, accommodative lag, peripheral refraction, choroidal thickness, aberrometry, pupil size, QoL, symptoms, compliance, safety	After 3 yrs: Adjusted SER progression: −0.60 D (High), −0.89 D (Medium), −1.05 D (SV). AL growth: 0.42 mm (High), 0.58 mm (Medium), 0.66 mm (SV). High add slowed progression by 43% vs SV. Dose-response observed. VA unaffected. Mild AEs.
Chen et al. [8]	China	Prospective, randomized controlled trial (single-center)	N=64 enrolled (DFSCL=32, SV=32); Completed=58 (DFSCL=28, SV=30); Age 8–12 yrs, mean 9.9 ± 1.2; Sex: DFSCL 17F/11M, SVS 20F/10M	DFSCL: −1.88 ± 0.79; SV: −1.95 ± 0.68	DFSCL: 24.21 ± 0.73; SV: 24.57 ± 0.66	MiSight 1-day DFSCL (CooperVision, omafilcon A, concentric +2.00 D defocus/correction zones; 60% water, 14.2 mm dia, 8.7 mm BC)	Single-vision spectacles (Kodak, aspheric)	Daily wear, gradually increased to 12–15 h/day; ≥6 days/week; monitored at each follow-up	12	Primary: AL (IOLMaster 700 SS-OCT), SER (cycloplegic autorefraction i-Trace & retinoscopy). Secondary: % rapid AL growth ≥0.4 mm/year, % progressive myopia ≥0.75 D/year, pupil size, vision/comfort, corneal status (slitlamp, Efron). Tools: IOLMaster 700, i-Trace, slitlamp Topcon SL7	DFSCL group: lower AL growth (0.23 ± 0.03 mm vs 0.33 ± 0.02 mm, P=0.004), no significant SER difference (−0.44 vs −0.53 D, P=0.308). Fewer rapid AL growth events (11% vs 40%). Mild initial blur, long-term dryness/itching; no serious AEs. Short-term AL reduction confirmed.
Zhang et al. [9]	Hong Kong & China	Randomized, double-masked, parallel-group RCT	N=167 enrolled (DISC3.5plus=87, SV=80); Completed=98 (DISC3.5plus=45, SV=53); Per-protocol=73; Age 8–13 yrs, mean 10.9 ± 1.9; ~60% female	DISC3.5plus: −2.40 ± 0.90; SV: −2.76 ± 1.09	DISC3.5plus: 24.65 ± 0.91; SV: 24.41 ± 0.85	DISC3.5plus lenses (Efrofilcon A, SiHy, monthly; central correction + concentric alternating distance & defocus zones; +3.50 D initial defocus, outer rings up to +6.00 D)	Single-vision SCLs (Efrofilcon A, same material)	Daily wear, ≥6 h/day; SV spectacles remainder of time	12	Primary: SER (cycloplegic autorefraction). Secondary: AL (IOLMaster 500), VA (ETDRS high/low contrast, near), stereopsis, accommodation, compliance, AEs	ITT: DISC3.5plus slowed SER at 6 mo (−0.25 vs −0.40 D, p=0.02) but not 12 mo (−0.44 vs −0.56 D; p=0.11). AL effect nonsignificant at 12 mo. Per-protocol: SER reduced at both 6 & 12 mo; AEs mild. Promising short-term efficacy; long-term limited by COVID-19 disruptions and dropout.
Walline et al. [10]	USA (Ohio State & Univ. of Houston)	Randomized, double-masked, 3-arm clinical trial	N=294 children 7–11 yrs, mean 10.3 ± 1.2; 60% female; SER −0.75 to −5.00 D, ≤1.00 D astigmatism	−2.39 ± 1.00	24.48 ± 0.81	Biofinity Multifocal “D” SCLs (CooperVision; Comfilcon A, monthly replacement; +1.50 D or +2.50 D add)	Biofinity single-vision SCLs	Monthly replacement; daily wear; 3-year trial	36	Primary: SER (cycloplegic autorefraction). Secondary: AL (Lenstar LS900), peripheral eye length, VA, accommodative lag, QoL (PREP-2), symptoms, slit-lamp, AEs	Baseline/methods paper; demographics and study design. No efficacy results. Highlights BLINK as the first RCT comparing +1.50 vs +2.50 D add in multifocal SCLs for myopia control.
Chamberlain et al. [14]	Multicenter: Portugal, UK, Singapore, Canada	Randomized, double-masked, parallel-group clinical trial	N=135 randomized (MiSight=70, Control=74); Completed=109 (MiSight=53, Control=56); Age 8–12 yrs (mean 10.1 ± 1.3); Sex ~50% male/female; ethnically diverse	MiSight: −2.02 ± 0.77; Control: −2.19 ± 0.81	MiSight: 24.42 ± 0.70; Control: 24.46 ± 0.66	MiSight 1-day DFSCL (CooperVision; omafilcon A, daily disposable, concentric dual-focus +2.00 D add)	Proclear 1-day (CooperVision; same material, monofocal)	Daily disposable, ≥10 h/day, ≥6 days/week	36	Primary: SER progression (cycloplegic autorefraction), AL growth (IOLMaster). Secondary: BCVA, near VA, comfort/satisfaction, wearing time, slitlamp grading, AEs	MiSight reduced SER progression by 0.73 D (59%) and AL growth by 0.32 mm (52%) vs control over 3 years. Strong AL-SER correlation (r = −0.90). High compliance, good acceptance. No serious AEs. Effective and safe long-term.
Mutti et al. [15]	USA (Ohio State Univ., Univ. of Houston)	Randomized, double-masked, 3-arm clinical trial	N=294, Age 7–11 yrs, mean 10.3 ± 1.2, 60% female; 26% Hispanic/Latino, 68% White. Randomized 1:1:1 +2.50 D add, +1.50 D add, SV	−2.39 ± 1.00; astigmatism <1.00 D	Not reported; AL progression used	Biofinity center-distance multifocal SCLs (CooperVision, +2.50 D add, +1.50 D add)	Biofinity single-vision SCLs	Daily wear, monthly replacement; monitored every 6 months	36	Primary: Axial & peripheral eye elongation (Lenstar LS900 at fovea ±20°, ±30°); cycloplegic refraction (Grand Seiko WAM-5500). Secondary: peripheral refraction, quadrant/meridian treatment effect	+2.50 D add lenses reduced axial elongation over 3 yrs (0.39 mm vs 0.63 mm SV, p<0.001). Greatest inhibition at the fovea. +1.50 D add intermediate, nonsignificant. Global defocus control is suggested.
Weng et al. [16]	China (AIER Hospital Group, Guangzhou)	Prospective, randomized, contralateral, cross-over clinical trial	N=95, 7–13 yrs (mean 10.8 ± 1.5), ~50% female; 3 groups: I bilateral SVCL, II EDOF vs SV, III MiSight vs SV	−1.99 ± 0.68	24.6 ± 0.8	Group II: EDOF CL (Aquamax, etafilcon A, daily, HOA manipulation). Group III: MiSight CL (Omafilcon A, dual-focus +2.00 D add)	Single-vision CL (1-day Acuvue Moist, etafilcon A)	Daily disposable, all waking hours; Stage 1 = 6 mo, then cross-over Stage 2 = 6 mo	12	Primary: SER (cycloplegic autorefraction), AL (Lenstar 900). Secondary: High/low contrast VA, comfort, compliance, AEs	Both MiSight & EDOF slowed progression vs contralateral SVCL: Stage 1: −0.16 D & −0.10 mm (EDOF), −0.16 D & −0.08 mm (MiSight). Stage 2: −0.20 D & −0.11 mm (EDOF), −0.08 D & −0.06 mm (MiSight).

Quality Assessment

Most studies demonstrated strong methodological quality. Large RCTs such as Chamberlain et al. [14], Mutti et al. [15], and Walline et al. [7] scored 27/28, and García-del Valle et al. [5] scored 26/28, reflecting robust designs and reporting. Other RCTs [1,4,8,9] scored 22-23, with minor limitations in external validity or confounding control. Observational and long-term studies [2,3,6] scored 20-21 due to reduced external validity and limited power, but all met acceptable standards, ensuring meaningful contribution to the evidence base (Table 2).

**Table 2:** Methodological quality assessment of myopia control contact lens studies Reporting (0–10) indicates the completeness of study reporting; External Validity (0–3) reflects the generalizability of the study population and settings; Internal Validity – Bias (0–7) assesses the risk of bias in study conduct; Internal Validity – Confounding (0–6) evaluates the control for potential confounding factors; Power (0–1) indicates whether the study was adequately powered to detect significant effects; Total (0–28) is the sum of all scores representing overall methodological quality. DF: dual-focus; SCL: soft contact lens; RCT: randomized controlled trial; EDOF: extended depth-of-focus; OK: orthokeratology; PAL: progressive addition lens

Study ID / Author	Reporting (10)	External Validity (3)	Internal Validity – Bias (7)	Internal Validity – Confounding (6)	Power (1)	Total (28)
Anstice & Phillips [1]	9	2	6	5	1	23
Santodomingo-Rubido et al. [2]	9	2	5	4	0	20
Santodomingo-Rubido et al. [3]	9	2	5	4	0	20
Lee et al. [4]	9	2	6	5	1	23
García-del Valle et al. [5]	10	3	6	6	1	26
Díaz-Gómez et al. [6]	9	2	5	4	1	21
Walline et al. [7]	10	3	7	6	1	27
Chen et al. [8]	9	2	6	5	1	23
Zhang et al. [9]	9	2	6	5	1	23
Walline et al. [10]	9	3	6	6	1	25
Chamberlain et al. [14]	10	3	7	6	1	27
Mutti et al. [15]	10	3	7	6	1	27
Weng et al. [16]	9	2	5	5	1	22
Yang et al. [17]	9	2	6	5	1	23

Effect on Spherical Equivalent Refraction Progression

Contact lenses consistently slowed myopic shift versus single-vision comparators, with magnitude depending on design, adherence, and follow-up. Dual-focus/center-distance multifocals (e.g., MiSight) showed the most consistent effects, reducing cumulative three-year SER progression by 0.40-0.73 D [14] (~59-69% relative slowing). High-add Biofinity (CooperVision, Inc., San Ramon, California, United States) multifocals in BLINK achieved 0.45 D less progression than single-vision [7]. Alternative designs (Esencia, MYLO, EDOF) showed favorable but more variable effects [5,6,16]. High-defocus lenses (DISC3.5plus) demonstrated adherence-dependent benefits [9], and orthokeratology slowed refractive progression over seven to 11 years [2,3]. Overall, SER progression was reduced by ~0.2-0.7 D over one to three years.

Effect on Axial Length Elongation

AL outcomes mirrored SER results. Dual-focus lenses reduced elongation by 40-60% over two to three years, with MiSight showing ~0.32 mm less growth [14] and high-add BLINK lenses ~0.23 mm less [7]. EDOF and Esencia lenses showed clinically meaningful reductions [6,16], while DISC3.5plus yielded smaller, non-significant differences [9]. Orthokeratology produced ~0.69 mm less cumulative growth over 11 years [2,3].

Effect on Visual Acuity and Performance

High-contrast visual acuity remained largely unchanged, with low-contrast reductions generally <1 line, not clinically meaningful [1,5,7,9,14]. Subjective metrics for clarity and comfort were favorable, though early adaptation occasionally caused near blur or dryness [8].

Effect on Compliance and Wear Time

Treatment efficacy depended on consistent daily wear (≈11-14 h/day, six to seven days/week) [7,14]. Studies with high discontinuation rates (33-50%) reported reduced benefits, though continuers maintained clear effects [9,16].

Effect on Safety and Adverse Events

No vision-threatening events were reported. Adverse events were mild and self-limited, including superficial punctate staining, papillary changes, and transient infiltrates, with similar incidence between myopia-control and single-vision lenses [2-7,9,14]. Orthokeratology showed no severe complications over long-term follow-up [2,3]. Overall, contact lens interventions were safe and well-tolerated in children.

## Conclusions

In summary, our findings, together with recent systematic reviews, support contact lens-based myopia control as both effective and safe in children. The strongest and most consistent evidence exists for dual-focus/high-add soft lenses, with complementary short-term support for EDOF designs and sustained, though methodologically qualified, benefits for orthokeratology. Minor visual performance trade-offs appear clinically acceptable for most patients. Early initiation, appropriate lens selection, and high adherence are key to maximizing real-world impact on axial growth and reducing lifetime myopia-related risks.
